# Supplementary material for: Observation of nanoscale magnetic fields using twisted electron beams
Source: Nat Commun. 2017 Sep 25;8:689. doi: 10.1038/s41467-017-00829-5 (PMC5613010; doi:10.1038/s41467-017-00829-5)
Supplement: Supplementary file 1 — Supplementary Information [file 41467_2017_829_MOESM1_ESM.pdf]

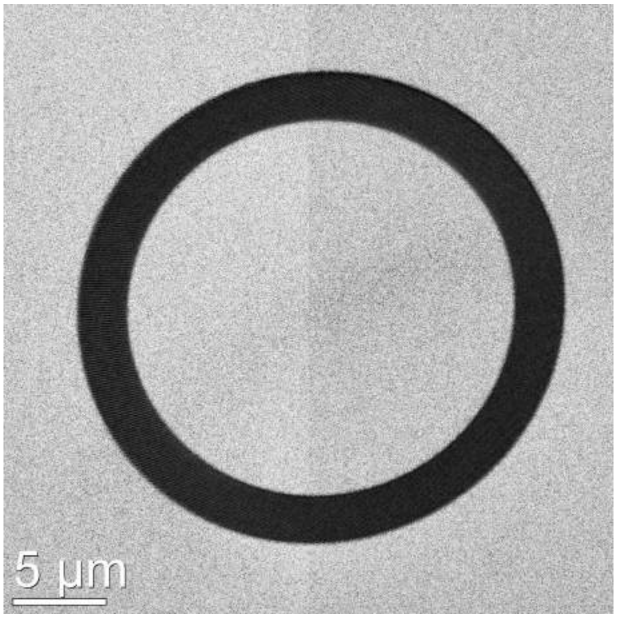

Supplementary Figure 1. **Image of the fabricated hologram.** The hologram, obtained using an energy filtered transmission electron microscope, is used to generate an electron beam of $\ell = 200$.

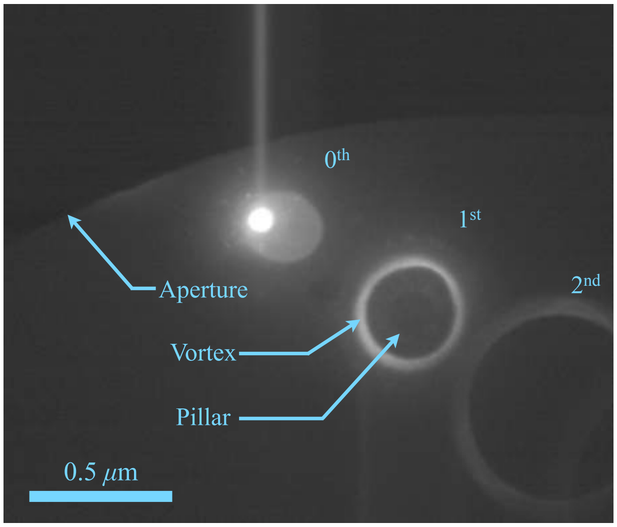

Supplementary Figure 2. **Example of the diffraction orders of the twisted electron beam with the magnetic pillar.** By defining the singular vortex core as the region where the intensity drops below 5% of its maximum, this area is enclosed in a radius that is 10-15% smaller than the beam radius ρ_m.

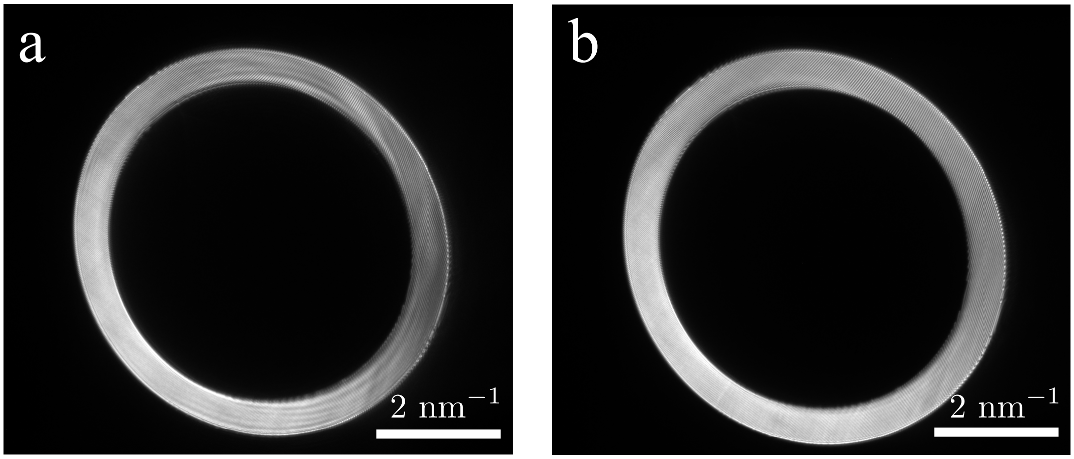

Supplementary Figure 3. **Interference pattern.** Interference pattern with (**a**) and without (**b**) the presence of the first magnetic pillar sample.

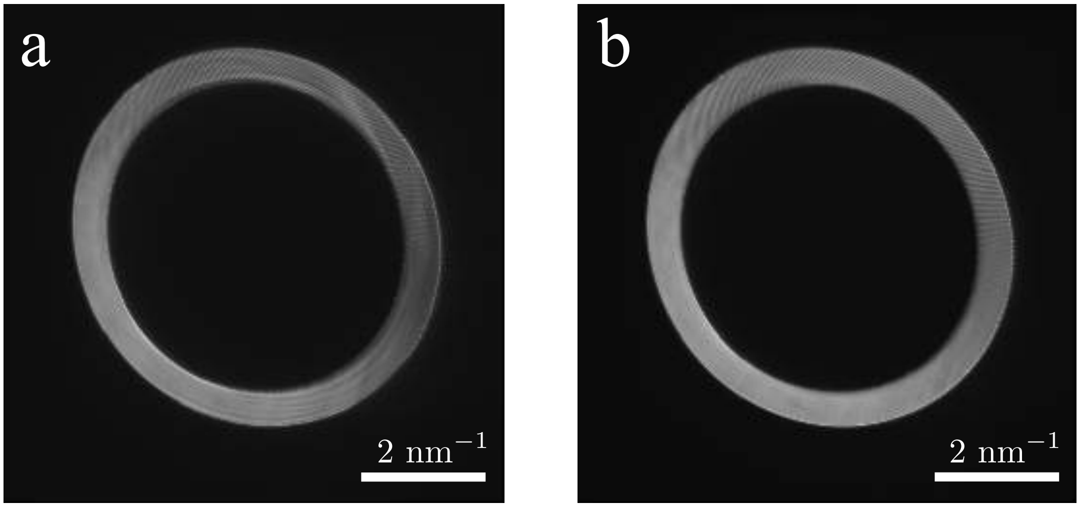

Supplementary Figure 4. **Interference pattern.** Interference pattern with (**a**) and without (**b**) the presence of the second magnetic pillar sample.

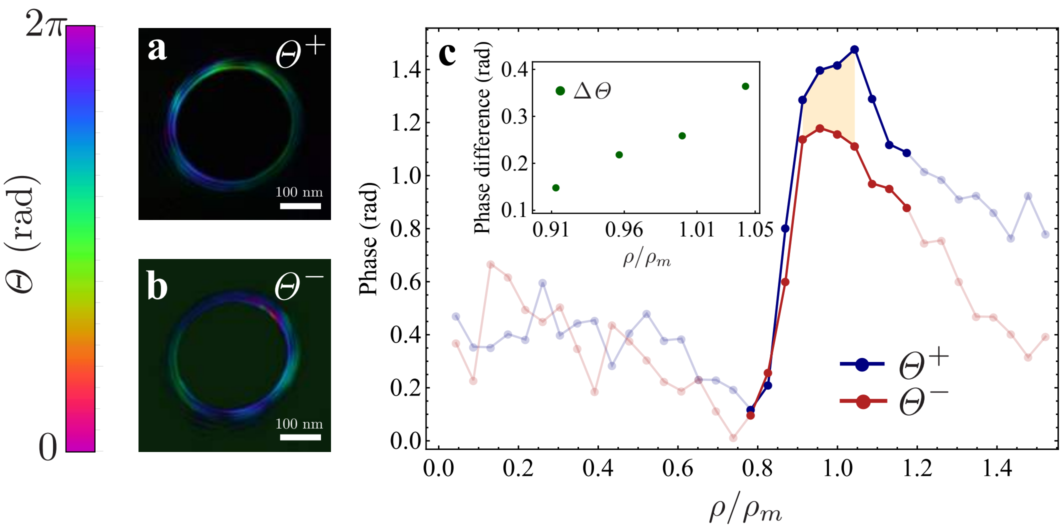

Supplementary Figure 5. **Experimentally observed phase shift of twisted electron beams upon interaction with the second sample containing a magnetic pillar**. **a** and **b** show the digitally reconstructed phase-shift for beams carrying OAM values of +200 and −200, respectively. **c** The points indicate the experimental data for measured relative phase changes. Red and blue colours are the measured phases for beams carrying positive Θ^{+} and negative Θ^{-} OAM value of 200, i.e. $\ell = \pm 200$. The relative phase $\Delta\Theta$ is illustrated by the beige shaded area. This data was evaluated for regions close to $\rho_m = 0.16\ \mu$m radius. Semi-transparent zones indicate regions where the probability of finding electrons is zero. The solid lines are obtained by interpolation.

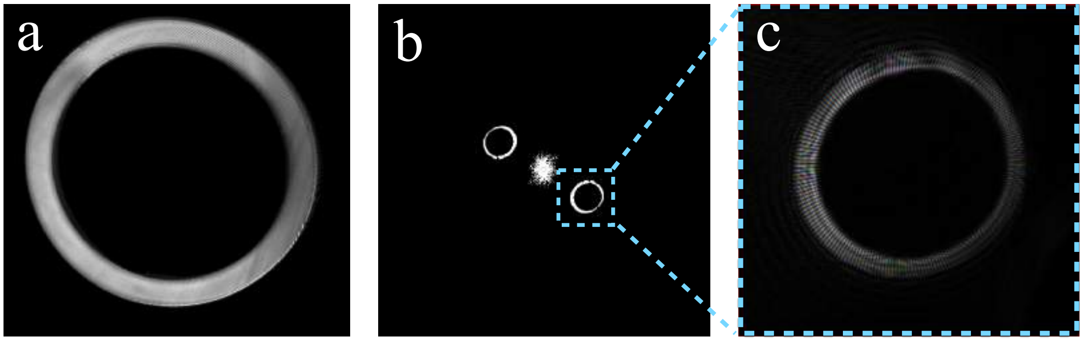

Supplementary Figure 6. **Extracting the magnetic field.** **a** Interference pattern of signal and reference beam. **b** Fourier transform of the interference. **c** Zoomed-in view of the first-order reconstructed signal beam in the Fourier transform of the interference.

SUPPLEMENTARY NOTE 1: DERIVATION OF RELATIVE PHASE BETWEEN POSITIVE AND NEGATIVE OAM COMPONENTS

Electron beams carrying OAM, i.e. twisted electron beams, possess a propagation dependent global phase which scales with the magnitude of its OAM value. This phase is known as the Gouy phase, and is given by the following expression:

$$\Theta_{\text{Gouy}} = (|\ell| + 1) \arctan\left(z/z_{\text{R}}\right), \qquad (1)$$

where ℓ is the OAM value of the electron, z is the propagation distance, $z_{\text{R}} = \pi w_0^2/\lambda_{\text{dB}}$ is the Rayleigh range and w_0 is a beam parameter given by the beam radius at the waist. Here, we consider that these twisted electrons pass around a magnetic dipole, as shown in Fig. 3, which we mathematically describe here as two magnetic monopoles separated by a distance d with opposite magnetic charge. Indeed, it has been shown that by passing through the first magnetic monopole, the twisted electron beam will see its OAM value, ℓ increase to $\ell + \nu$, where ν is the topological charge due to the Aharonov-Bohm phase. Subsequently passing through the second monopole, the twisted electron beam's OAM value is decreased from $\ell + \nu$ back to ℓ. It is our goal to calculate the relative phase between a twisted electron beam with an OAM value of ℓ and $-\ell$ due to the difference in Gouy phase between two monopoles (upon interaction with a magnetic dipole).

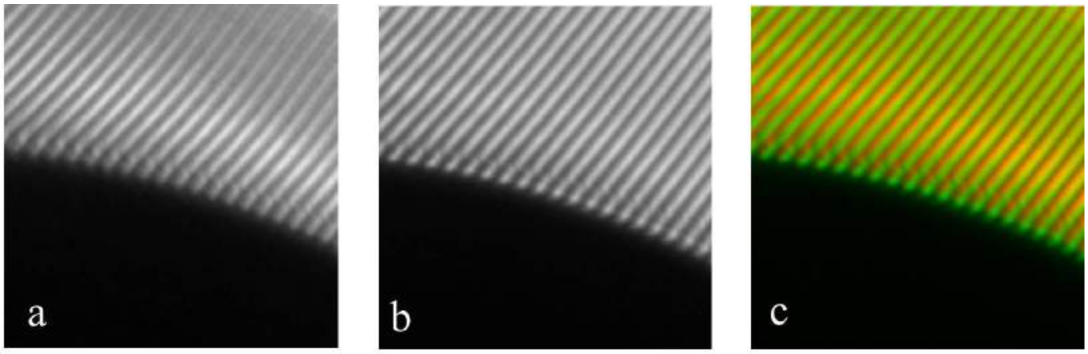

Supplementary Figure 7. **Interference fringes.** Comparison of a small cut-out of interference fringes using a+200$\hbar$ signal beam. **a** Signal beam on the first pillar described in the main text. **b** Signal beam far from the pillar. **c** A superposition image of the fringes after the alignment procedure where the red corresponds to **a** and green corresponds to **b**.

We consider a propagation distance of $z = d$ that is much smaller than the Rayleigh range ($d/z_R \sim 10^{-5}$ for our experimental parameters). For a Bessel-Gauss beam, the Rayleigh range, z_R, may be expressed in terms of the radius of maximum intensity, ρ_m:

$$z_R \approx \frac{\pi\rho_m^2}{|\ell|\lambda_{dB}} \qquad \text{for non-zero } \ell. \tag{2}$$

Assuming the above relation, for a short range propagation distance of d, i.e. $d \ll z_R$, the Gouy phase is given by the following expression

$$\Theta_{Gouy}(\ell) \approx (|\ell| + 1)\frac{d}{z_R}, \tag{3}$$

$$\approx \frac{d\lambda_{dB}}{\pi\rho_m^2}|\ell|^2. \tag{4}$$

We can now evaluate the Gouy phase for twisted electron beams with positive and negative OAM values ℓ and $-\ell$,

$$\Theta^+ \approx \frac{d\lambda_{dB}}{\pi\rho_m^2}(\ell + \nu)^2, \tag{5}$$

$$\Theta^- \approx \frac{d\lambda_{dB}}{\pi\rho_m^2}(-\ell + \nu)^2. \tag{6}$$

Thus, the relative phase between two beams carrying opposite values of OAM upon interacting with the magnetic dipole is

$$\Delta\Theta \approx \left(\frac{d\lambda_{dB}}{\pi\rho_m^2}\right)4\ell\nu. \tag{7}$$

SUPPLEMENTARY NOTE 2: ALTERNATIVE DERIVATION.

The induced Gouy phase can be derived upon certain approximations without invoking the action of magnetic monopole. Since the magnetic pillar is spatially localised, $d/z_R \sim 10^{-5}$ and the propagation of the twisted electrons can be examined in three different regions: (i) free-space propagation before the pillar, (ii) during interaction with the pillar, and (iii) free-space propagation after the pillar. For cases (i) and (iii), i.e. before and after the pillar, the magnetic field and magnetic flux seen by the twisted electrons vanish quickly, while the twisted electrons see a fairly constant magnetic flux in the second region. Therefore, the dynamics of the electron wavefunction for distances far from the pillar, $\psi_i(\mathbf{r})$ and $\psi_{iii}(\mathbf{r})$, and close to the pillar $\psi_{ii}(\mathbf{r})$ can be

described by the following equations

$$\begin{cases} \left(\frac{1}{\rho}\partial_\rho(\rho\,\partial_\rho) + \frac{1}{\rho^2}\partial_\varphi^2 - 2ik\partial_z\right)\psi_{\mathrm{i}}(\mathbf{r}) = 0 & z \ll -d/2 \\ \left(\frac{1}{\rho}\partial_\rho(\rho\,\partial_\rho) + \frac{1}{\rho^2}(\partial_\varphi - i\nu)^2 - 2ik\partial_z\right)\psi_{\mathrm{ii}}(\mathbf{r}) = 0 & |z| \lesssim d/2 \\ \left(\frac{1}{\rho}\partial_\rho(\rho\,\partial_\rho) + \frac{1}{\rho^2}\partial_\varphi^2 - 2ik\partial_z\right)\psi_{\mathrm{iii}}(\mathbf{r}) = 0 & z \gg d/2 \end{cases}, \tag{8}$$

where $\nu = e\Phi/(2\pi\hbar)$ is the dimensionless magnetic flux seeing by the twisted electrons, $k = \sqrt{2m_e\mathcal{E}}/\hbar$ is the electron wavenumber, and ρ, φ and z are the cylindrical coordinates. Neglecting higher-order radial modes and keeping the $p = 0$ mode, the generated twisted electrons in the first region can be expressed by the following expression in the Laguerre-Gauss basis:

$$\psi_{\mathrm{i}}(\mathbf{r}) = \mathcal{A}_\ell(\rho, z)\, e^{i\ell\varphi} e^{i(|\ell|+1)\arctan(z/z_{\mathrm{R}})}, \tag{9}$$

where $\mathcal{A}_\ell(\rho, z)$ is the amplitude of the Laguerre-Gauss mode [1]. Upon interaction with the pillar, a constant magnetic flux, only the radial probability density distribution of the electron beam will be altered (the azimuthal index does not change). Thus,

$$\psi_{\mathrm{ii}}(\mathbf{r}) = \mathcal{B}_{\ell-\nu}(\rho, z)\, e^{i\ell\varphi} e^{i(|\ell-\nu|+1)\arctan(z/z_{\mathrm{R}})}, \tag{10}$$

where $\mathcal{B}_{\ell-\nu}(\rho, z)$ is the new electron amplitude function. Since the $\mathcal{B}_{\ell-\nu}(\rho, z)$ is not an eigenfunction of the free-space radial operator, the beam amplitude changes back to the conventional form after the pillar, that is

$$\psi_{\mathrm{iii}}(\mathbf{r}) = C_\ell(\rho, z)\, e^{i\ell\varphi} e^{i(|\ell|+1)\arctan(z/z_{\mathrm{R}})}. \tag{11}$$

Continuity of the electron wavefunction in space (region (i), (ii) and (iii)) dictates the relation between $\mathcal{A}_\ell$ and $\mathcal{B}_{\ell-\nu}$, and $\mathcal{B}_{\ell-\nu}$ and C_ℓ. The twisted electrons gain a phase of $(|\ell - \nu| + 1)\arctan(z/z_{\mathrm{R}}) \approx (|\ell - \nu| + 1)(d/z_{\mathrm{R}})$ upon interacting with the pillar. Substituting the Rayleigh range z_{R} by the beam radius (see Eq. (2)) results in the same phase shift as that previously derived in Supplementary Note 1.

SUPPLEMENTARY NOTE 3: EXTERNAL MAGNETIC OR ELECTRIC FIELD

An external in-plane magnetic field may induce a phase to the electron beam, which is typically constant across the electron beam transverse plane. The implemented differential phase technique removes these effects because they do not depend on the OAM value of the signal beam. As for the vertical magnetic field, the main contribution comes from the magnetic objective lens. In our experimental apparatus, the magnetic objective lens is almost turned off (LOW MAG configuration). Nevertheless, a small field for a long propagation distance can induce a rotation, and therefore a global OAM-dependent phase term, to the electron wavefunction. Since the induced phase is proportional to B and increases linearly, differential measurements can be used to remove such effects. In fact, the total relative phase difference is given by the expression

$$\Delta\Theta = \frac{1}{2\pi}\int \left(\left[\Gamma^+\left(B_{\mathrm{ext}} + B_{\mathrm{pillar}}\right) - \Gamma^+\left(B_{\mathrm{ext}}\right)\right] - \left[\Gamma^-\left(B_{\mathrm{ext}} + B_{\mathrm{pillar}}\right) - \Gamma^-\left(B_{\mathrm{ext}}\right)\right]\right) \mathrm{d}\varphi, \tag{12}$$

where Γ^+ and Γ^- are the phase of the twisted electrons carrying OAM values of $+\ell$ and $-\ell$, respectively, B_{pillar} is the magnetic field of the pillar, and B_{ext} is the external magnetic field. Γ^+ and Γ^- are assumed to form the following topological structures

$$\Gamma^\pm = \pm\ell\varphi + \Gamma_0^\pm, \tag{13}$$

where $\Gamma_0^\pm$ are constant global phases. The induced phase is a linear function of magnetic field. Thus, we can write $\Gamma^+\left(B_{\mathrm{ext}} + B_{\mathrm{pillar}}\right) = \Gamma^+\left(B_{\mathrm{ext}}\right) + \Gamma^+\left(B_{\mathrm{pillar}}\right)$. Therefore, the contribution from $\Gamma^+\left(B_{\mathrm{ext}}\right)$ will be omitted from (12) and does not play a role in our analysis. This means that the measurement should equally work even when the objective lens is turned on.

SUPPLEMENTARY NOTE 4: EXTRACTING THE MAGNETIC FIELD

The process of extracting the induced phase due to the longitudinal magnetic field is complicated, because any effect independent on/from the shape of the twisted electron beam needs to be removed. For example, lensing effects from the pillar field acting as a magnetic lens should be nearly independent on OAM. A typical image of the two beam interference pattern is shown

in Supplementary Figure 6-a. The Fourier transform of the interference pattern produces a complex phase image of the beam at the level of the sample. Supplementary Figure 6-b shows a typical Fourier transform, while Supplementary Figure 6-c is the phase image of a single diffraction order. In Supplementary Figure 6-c the phase is indicated by a colour scale, as in the main text, while the image brightness is proportional to the Fourier intensity.

The phase is clearly dominated by the azimuthal ramp typical of a vortex beam. The global phase term acquired by the vortex beam upon interaction with the longitudinal magnetic field, which is theoretically constant over the vortex ring, can only be extracted as a difference from a reference vortex Fourier transform. In fact, the process was applied to four experimental images; $+\ell$ OAM-carrying twisted electron beams with and without interacting with the magnetic pillar, and $-\ell$ OAM-carrying twisted electron beams with and without interacting with the magnetic pillar. Specifically, the 'without' data was recorded after moving the magnetic pillar specimen far (microns) away from the reference and signal beam. The relative phase between 'with' and 'without' pillar images was separately calculated for both electron beams. The alignment was carried out using an interactive graphical procedure of STEMCELL [2] that permits to adjust the relative shift between interferometric images while looking at the Fourier phase difference, and looking for a nearly constant phase difference. Since the phase difference is nearly flat, no phase unwrapping is required. The phase difference is averaged over all azimuthal angles, obtaining data that are independent of small pillar shifts.

In this process, it is possible to remove physical linear phases. For example, in order to switch from the $+200\hbar$ to $-200\hbar$ signal beam, the reference beam switches from one side of the pillar to the other. If the interaction of the reference beam with the long-ranged in-plane component of the magnetic field is slightly different on each side, one could expect small and unequal distortions in the interference fringes due to the difference in the perturbed reference wave [3]. We assume such distortions are small in this work, as the pillar's magnetic field is highly cylindrically symmetric and the transverse momentum (beam tilt) of the reference beam that might allow for some sensitivity of the reference beam to the longitudinal field is small.

We are developing more robust alignment techniques to deal with this problem. In particular, averaging of the phase with some weighting by the intensity or amplitude allows for more robust averaging of phase distributions. A reference phase image formed with a straight diffraction grating can independently quantify phase variations due to electrostatic and in-plane magnetic fields even in the case of imperfect alignment [4]. Although these additional steps are outside the scope of this work, we note that the azimuthal average of the phase as a function of radius (see Fig. **5**) washes out the effect of astigmatism or small misalignments and avoids some of the problems that can arise with an unweighted average of phase in low-amplitude regions.

To simplify the phase representation, in the main text we represented the relative phase with a Hue colour scale. The brightness of the colours is proportional to that of one of the two electron vortices. The phase was explicitly calculated in the two cases and subtracted as described in the main text. The magnetic field in the pillar was calculated using the equation given in the main text (and Supplementary Note 1) for the flux, assuming that the magnetic field inside the pillar is constant.

SUPPLEMENTARY REFERENCES

[1] Bliokh, K. Y., Schattschneider, P., Verbeeck, J. & Nori, F. Electron vortex beams in a magnetic field: A new twist on landau levels and aharonov-bohm states. *Physical Review X* **2**, 041011 (2012).

[2] Grillo, V. & Rossi, F. Stem_cell: A software tool for electron microscopy. part 2 analysis of crystalline materials. *Ultramicroscopy* **125**, 112–129 (2013).

[3] Matteucci, G. *et al.* Electron holography of long-range electric and magnetic fields. *Journal of applied physics* **69**, 1835–1842 (1991).

[4] Yasin, F. S., Harvey, T. R., Chess, J. J., Pierce, J. S. & McMorran, B. J. Development of stem-holography. *Microscopy and Microanalysis* **22**, 506–507 (2016).
